# Towards Levelling the Playing Field for Prospective Overseas Applicants to Medical Schools in the United Kingdom

**DOI:** 10.7759/cureus.32786

**Published:** 2022-12-21

**Authors:** Setthasorn Zhi Yang Ooi, Artemis Mantzavinou

**Affiliations:** 1 School of Medicine, Cardiff University, Cardiff, GBR; 2 Medicine, Barts and the London School of Medicine and Dentistry, Queen Mary University, London, UK, London, GBR

**Keywords:** brexit, prospective applicants, international, overseas, tuition fees, widening participation

## Abstract

Many factors are taken into consideration when students apply to pursue medicine in the United Kingdom. For overseas applicants, the tuition fees of the medical course are a significant factor, particularly for those from lower socioeconomic backgrounds. The reasons why the fees in some medical schools are significantly higher than in others are unclear. Transparency on the use of the tuition fees as well as providing overseas medical students, which now include European students post-Brexit, with more financial support in their studies would be imperative. This is to ensure students are able to choose their medical schools based on more important factors such as the student-curriculum fit.

## Editorial

Medical schools across the United Kingdom (UK) hold a prestigious reputation worldwide. In 2021, there were 28,690 applicants for only 9,500 medical school places, raising the question of how the most suitable candidates are selected [1]. The competitiveness of applying to a UK medical school as an overseas applicant is further compounded by the small quota of international student intake, capped at 7.5% per UK medical school [2]. Given the high status of medical schools in the UK along with the competitive application process required, implementing a fair and transparent selection process is required to ensure equitable access to medical school places.

Applying to medical universities in the UK is a complex process, with a variety of factors to be considered by i) the medical school (e.g. the skills and knowledge of the applicant) and ii) the student (e.g. their preference for medical schools along with personal circumstances such as their capacity for funding). For the former, the assessment of selection by the medical schools is based on a ‘predictive paradigm’, where the intention is to identify candidates who are predicted to successfully complete training and become competent doctors [3]. High academic achievement is demonstrated through applicants’ merits, and school grades are a minimum entry requirement. Some of the most common required grades are predicted and actual A Levels, General Certificate of Secondary Education (GCSE) grades and International Baccalaureate scores. Depending on the preferences of medical school applicants, aptitude test scores (e.g. the University Clinical Aptitude Test (UCAT) and BioMedical Admissions Test (BMAT)) also form part of the application process. The selection process is aided by applicants’ personal statements demonstrating extracurricular interests and achievements along with their interview performance assessing essential healthcare and interpersonal skills (e.g. teamwork, communication, and decision-making).

Applicants can apply to a maximum of four medical schools, as indicated on the Universities and Colleges Admission Service (UCAS) application portal. Many factors are taken into consideration when students shortlist their four medical schools of choice. These factors include the student’s preference for course structure (e.g. traditional versus case/problem-based learning versus integrated), the strength of application (i.e. grades, test scores and personal statement), length of the course (5 years versus 6 years), pedigree/ranking of the university as well as non-academic factors such as the location of the university, social life opportunities and living cost. It is pivotal that students consider these factors and reflect on their personal abilities and goals to ensure they optimise their learning and maximise their potential during their time in medical school. However, for overseas applicants, in particular, there is another factor that plays a highly influential role in their decision-making process; that is, tuition fees. The tuition fees for overseas applicants vary greatly across medical schools. For example, studying medicine at Newcastle University costs £38,400 per academic year [4] in comparison to Cambridge University where the course costs £63,990 per year [5] for the 2023 entry. This is further compounded by the implementation of the additional cost of training (ACT) levy, which imposes an added £10,000 per year on overseas medical students studying in medical schools in Scotland and Northern Ireland [2]. Overseas medical students are also subjected to annual inflation of 1-2% as per the Retail Price Index [2]. In addition to the tuition fees, the living costs also vary across different cities in the UK. Of note, overseas students are not considered eligible to claim available student loans, such as the UK government’s tuition fee and maintenance and postgraduate loans. 

This large amount of fees can place enormous financial pressure on overseas applicants, especially those from lower socioeconomic backgrounds when choosing their medical schools. This may render other said factors in the decision-making process less important. Regardless of the students' academic merits or qualifications, the applicants' choice of medical school is limited at the outset by their families' financial capabilities - a factor that students themselves have almost no control of. This means that students from lower socioeconomic backgrounds receiving limited or no external financial support would choose a medical school based on affordability as opposed to a medical school that they believe would help them maximise their potential as future doctors.

The reasons why the tuition fees in some medical schools are significantly higher than in others are unclear. Medicine should be open to people from all socioeconomic statuses, ethnicities and backgrounds, regardless of where they come from. Enhancing transparency on how the funds of overseas tuition fees are allocated will be useful in providing overseas applicants with a better understanding of whether medical schools with higher tuition fees provide more benefits to their students. This will also enable prospective applicants to make a more informed decision if they wish to apply to a more expensive medical school for those extra opportunities.

To the best of our knowledge, there have been no reports to date that discuss the issue of the variation of overseas medical students’ fees and its impact on medical school choice for prospective applicants. Furthermore, since Brexit, these points discussed now apply to applicants from Europe too [4]. Hence, the impact of the variation in the fees will extend to more prospective applicants, further compounding the issue and going against the UK’s mission to widen the participation of the underprivileged in medicine. It may be useful if medical schools across the board could offer more financial support to students such as offering merit-based scholarships or loans with a contract where the graduates are legally bound to serve the National Health Service (NHS) for a number of years to repay the loan.

This article is a call to action for stakeholders such as medical school governing bodies and policymakers to consider these issues and to identify ways to improve the accessibility of medical education to students of all backgrounds. We hope that this is a step forward to ensuring that the best and the brightest of students can access the medical schools of their choice regardless of their financial background.
